# Implementation Timeframes for the Addition of New Conditions to Newborn Bloodspot Screening Programmes: A Scoping Review

**DOI:** 10.3390/ijns11040106

**Published:** 2025-11-14

**Authors:** Margaret M. Brennan, Aoife O’Connell, Loretta O’Grady, Mohamed Elsammak, Jennifer J. Brady, Paul Marsden, Heather Burns, Abigail Collins

**Affiliations:** 1 Child Health Public Health, National Healthy Childhood Programme, Health Service Executive Area Office, Arden Road, Tullamore, Co. Offaly, Ireland; 2 National Newborn Screening Laboratory, Children’s Health Ireland at Temple Street, Temple Street, Dublin 1, Ireland; 3 UCD School of Medicine, UCD Health Sciences Centre, University College Dublin, Belfield, Dublin 4, Ireland

**Keywords:** implementation, timeframes, newborn screening, bloodspot, severe combined immunodeficiency, spinal muscular atrophy

## Abstract

Severe combined immunodeficiency (SCID) and spinal muscular atrophy (SMA) are being added to the Newborn Bloodspot Screening (NBS) programme in the Republic of Ireland. To support this expansion, we conducted a scoping review to identify reported timeframes for implementing national, regional or state-wide expanded NBS programmes. We performed a scoping review of the literature published between 2015 and 2025. Eligible articles described the timeframes for implementation of expanded NBS programmes for SCID, SMA or additional metabolic conditions. Sources included PubMed, Embase, citation searching, the International Journal of Neonatal Screening and grey literature. A narrative synthesis was undertaken. Fourteen articles met the inclusion criteria, describing the addition of new conditions—SCID (N = 7), SMA (N = 4), or multiple conditions (N = 3) to expanded NBS programmes in the United States (US), Europe (Belgium, Catalonia, the Czech Republic, Estonia, Germany, Norway, Poland, Portugal, Slovakia, Slovenia, Sweden, and Tuscany), Hong Kong and New Zealand. In most jurisdictions, the implementation of NBS programmes for new conditions took two to six years. The implementation of NBS for new conditions requires considerable time and coordinated efforts. Further research providing greater detail on the specific implementation steps, along with associated timelines, would provide valuable guidance for jurisdictions aiming to expand NBS programmes globally.

## 1. Introduction

As of June 2025, nine conditions are screened for through the National Newborn Bloodspot Screening (NBS) Programme in the Republic of Ireland. These are phenylketonuria, homocystinuria, classical galactosaemia, maple syrup urine disease, congenital hypothyroidism, cystic fibrosis, glutaric aciduria type 1, medium-chain acyl-CoA Dehydrogenase deficiency, and adenosine deaminase deficiency severe combined immunodeficiency [1].

In 2023, following health technology assessments conducted by the Health Information and Quality Authority [2,3], and recommendations from the National Screening Advisory Committee (NSAC), the Minister of Health approved the addition of Severe Combined Immunodeficiency (SCID) and Spinal Muscular Atrophy (SMA) to the Irish NBS programme. Further expansion of NBS is a priority workstream for the NSAC in Ireland, with over 30 additional conditions under consideration for future inclusion [4].

Preparations for implementation of SCID and SMA screening are underway within the National Healthy Childhood Programme, who have clinical governance for NBS in Ireland. As part of these preparations, this review aims to identify evidence on implementation timelines from jurisdictions that have successfully integrated molecular screening for SCID and SMA into NBS. We also sought to identify published evidence from jurisdictions that have added additional metabolic conditions detectable by tandem mass spectrometry, as more conditions may be added to the Irish NBS programme in the coming years.

## 2. Materials and Methods

### 2.1. Study Design

This is a scoping review reported in accordance with the Preferred Reporting Items for Systematic reviews and Meta-Analyses extension for Scoping Reviews checklist [5]. A scoping review approach was chosen due to the anticipated heterogeneity in study methods and disciplinary perspectives. This design allows for a comprehensive synthesis of existing evidence to inform practice, programs, and policy, while also identifying gaps to guide future research priorities [5,6]. We did not pre-register a protocol for this review.

### 2.2. Eligibility Criteria

Studies were eligible for inclusion if they met the following criteria, structured according to the Population, Intervention, Comparison, Outcome and Study type (PICOS) framework, and were published within the last ten years (2015–2025):

- Population: Newborns in high-income countries.
- Intervention: National, regional or state-level implementation of expanded bloodspot screening for SCID, SMA, or metabolic conditions during the newborn period [7].
- Control: Not applicable.
- Outcome: Timeframe for implementation. There is considerable heterogeneity in how this outcome is reported across the literature. Some publications describe timeframes from the point of view of governmental or regulatory approval, or pilot study initiation, while others mention earlier preparatory and political processes. To address this transparently, we report the timing associated with specific steps as described in each publication, where available.
- Study design: Evaluation of the implementation of a real-world screening programme.

Studies were excluded if they focused solely on pilot programmes or if a more recent publication was available with more comprehensive information for a given jurisdiction. The search was limited to English-language publications.

### 2.3. Information Sources

We searched PubMed, on the 31st of January 2025, for studies published between 2015 and 2025. Additional references were identified through snowball reference searching. Since the International Journal of Neonatal Screening (IJNS) was considered a key source, we hand-searched articles published within the last six months (July 2024–January 2025: IJNS Volume 10, Issues 3 and 4) to account for potential delays in time-to-indexing in PubMed [8]. A second database, Embase, was searched on the 6th of May 2025.

We also searched the US Health Resources and Services Administration (HRSA) and the United Kingdom’s (UK) official governmental website for grey literature on the implementation of SCID and SMA screening. The US was included due to its earlier adoption of SCID and, subsequently, SMA screening, making it a likely source of key information on implementation. The UK was selected for its known work on a pilot initiative. Both countries were also chosen for the accessibility of English-language materials.

### 2.4. Search

A comprehensive search strategy linked using Boolean operators was developed to capture the literature on implementation timelines for expanded newborn bloodspot screening programmes, provided below:

((((implement* OR implementation OR adopt* OR rollout) AND (newborn OR “new-born” OR neonate OR neonatal)) AND (“bloodspot screen” OR “Guthrie test” OR “heel prick” OR “heel-prick” OR screen* OR screening OR “tandem mass spectrometry”))) AND (“severe combined immunodeficiency” OR SCID OR “spinal muscular atrophy” OR SMA OR phenylketonuria OR “maple syrup urine disease” OR homocystinuria OR tyrosinemia OR “5-oxoprolinuria” OR “glutathione synthetase deficiency” OR citrullinemia OR “argininosuccinic aciduria” OR argininemia OR “short chain acyl-CoA dehydrogenase deficiency” OR “isobutyryl-CoA dehydrogenase deficiency” OR “glutaric aciduria” OR “multiple acyl-CoA dehydrogenase deficiency” OR “Medium/Short chain L-3-hydroxyacyl-CoA dehydrogenase deficiency” OR “medium chain acyl-CoA dehydrogenase deficiency” OR “Long chain 3 hydroxyacyl-CoA dehydrogenase deficiency” OR “trifunctional protein deficiency” OR “Very long chain acyl-CoA dehydrogenase deficiency” OR “carnitine palmitoyl transferase deficiency” OR “carnitine/acylcarnitine translocase deficiency” OR “carnitine uptake defect” OR “propionic acidemia” OR “methylmalonic acidemia” OR “malonic aciduria” OR “multiple carboxylase deficiency” OR “3-hydroxy 3-methylglutaric-CoA lyase deficiency” OR “3-methylcrotonyl-CoA carboxylase deficiency” OR “3-methylglutaconic aciduria” OR “2-methylbutyryl-CoA dehydrogenase deficiency” OR “isovaleric acidemia” OR “glutaric acidemia” OR “beta-ketothiolase deficiency”).

### 2.5. Data Charting Process

The literature search results were imported into Covidence, a systematic review management software [9], which performed automatic deduplication. A single reviewer performed title and abstract screening, then full text review based on the pre-defined eligibility criteria.

### 2.6. Data Items

The following data were extracted: author, year, country/region, study design, condition screened for, implementation steps and associated timeframes (where available) and time taken to implement new NBS programmes.

After identifying studies reporting on the primary review outcome (implementation timeframes) we decided, post hoc, to extract additional data on challenges and facilitators encountered when implementing SCID and SMA screening, recognising the relevance of these factors for current and future NBS programme expansions in Ireland and other jurisdictions.

### 2.7. Synthesis of Results

Narrative synthesis was performed.

## 3. Results

### 3.1. Selection of Sources of Evidence

**Figure 1:**
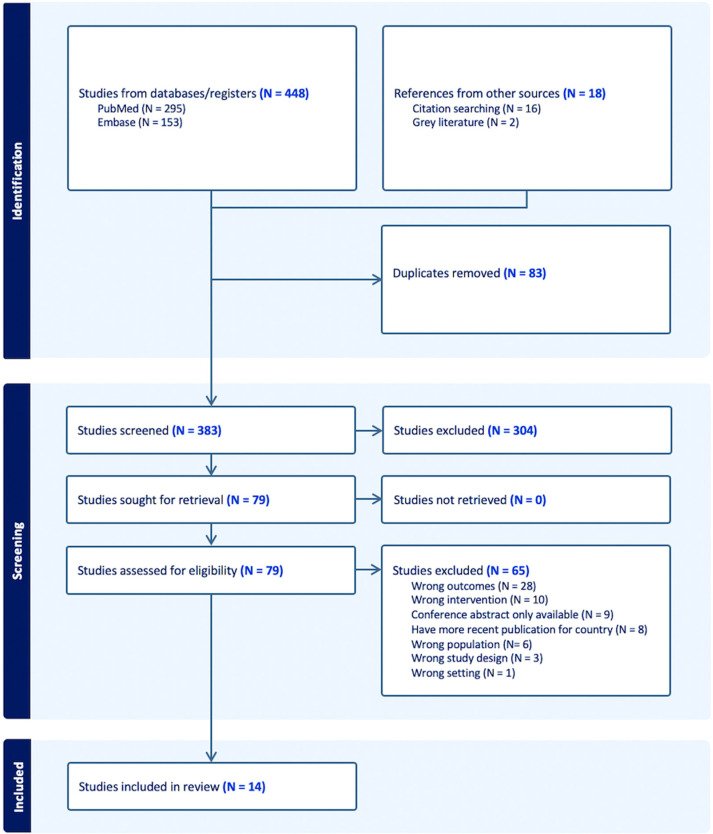
Flowchart depicting study identification and screening process.

We identified 466 publications, with 448 from PubMed and Embase and 18 found through citation searching and grey literature, with 83 duplicates. No additional studies were identified by hand-searching IJNS publications. After title and abstract screening, 304 studies were excluded. We then reviewed 79 full-text articles, excluding 65 that did not meet eligibility criteria in terms of outcomes, interventions, population, or study design, or because a more detailed and recent publication for that jurisdiction was available. Ultimately, 14 publications were included in the review (Figure 1).

### 3.2. Characteristics of Sources of Evidence

Seven publications (six peer-reviewed and one grey) described the addition of SCID to NBS programmes [10,11,12,13,14,15]. Four (three peer-reviewed and one grey) focused on SMA [16,17,18,19]. Three reported on the addition of multiple conditions [20,21,22].

One publication reported on multiple countries (Belgium, the Czech Republic, Estonia, Germany, Poland, Portugal, Slovakia and the US) [18], while three focused on the US only [19,20,23]. Seven described nationwide NBS programmes in six countries: New Zealand [10], Slovenia [22], Germany [12,16], Hong Kong [21], Norway and Sweden [11,14]. Three focused on regional programmes in Southern Belgium [24], Tuscany and Catalonia [13,15].

### 3.3. Narrative Synthesis 

Findings identified within the peer-reviewed literature are summarised in Table 1. Reported timeframes for the implementation of official NBS programmes for SCID ranged from eleven months (Catalonia) to eleven years (Germany) [12,13]. However, most studies indicated that the implementation of national, regional or state-level SCID screening programmes required between two and six years (Norway, New Zealand, Sweden, Tuscany, and US states) [10,11,14,15,20]. For SMA, implementation timeframes ranged from one to four years (Poland, US, Estonia, Germany, Portugal, Slovakia, Czech Republic and Belgium) [16,18,20,24]. Reports from Hong Kong and Slovenia, where NBS for multiple new conditions was implemented, indicated implementation timelines of approximately six years from pilot studies to national programmes [21,22].

### 3.4. Grey Literature Search

Governmental websites from the US and UK were searched for information on timelines for the implementation of screening programmes for SCID and SMA. Two relevant grey literature reports were identified from the US, but no reports were found for the UK. According to blog posts from the UK National Screening Service, SCID and SMA are not currently recommended as part of the NHS Newborn Blood Spot Screening Programme [25,26]. However, an in-service evaluation of SCID screening within live NHS services is underway [27], and planning has commenced for a similar evaluation of SMA [28]. The results of these evaluations are expected to inform whether SCID and SMA should become part of the NHS Newborn Screening Programme [27,28]. Results from the grey literature search are provided in Table 2.

### 3.5. Challenges and Facilitators in Implementing Screening for SCID and SMA

Challenges and facilitators encountered during the implementation of screening for SCID and SMA were reported in eight publications included in this review. Four reported on SCID and five on SMA (one publication reported on both SCID and SMA). A common challenge for both SCID and SMA was the need for dedicated lab space, agreed workflows, new equipment and expertise in molecular technologies. Other challenges and facilitators are detailed below in Table 3.

## 4. Discussion

### 4.1. Summary of Evidence

We identified twelve peer-reviewed and two grey literature publications reporting on timeframes associated with the national, regional or state-level implementation of expanded NBS programmes in the US [18,19,20,23], Europe [11,12,13,14,15,16,18,24], Hong Kong [21] and New Zealand [10].

The identified publications varied in how they described implementation timeframes, and this outcome was rarely a primary focus. Most reported timelines began with pilot studies [11,13,14,15,16,18,21,22,24], while others referenced the point of inclusion in screening panels or governmental approval [10,13,20]. One mentioned earlier political processes [12]. Despite these differences, most publications indicate that establishing new national, regional, or state-level newborn screening programs typically takes between two and six years.

The most comparable studies to the Irish context—where the NSAC and Minister for Health have recommended adding SCID and SMA to the NBS programme—appear to be those by Singh et al., (US) [20], Heather et al., (New Zealand) [10] and Argudo-Ramirez et al., (Catalonia) [13]. In the US, average implementation times within state-level programmes were 4.3 years for SCID screening, 2.1 years for SMA screening, 2.7 years for CCHD, 4 years for Pompe, 3.2 years for MPS-I and 3.5 years for X-ALD [20]. However, it is important to note that the timelines for implementing SMA screening in the US were supported by extensive preparatory work conducted by the Centers for Disease Control and Prevention in laboratory settings, which began in 2013, five years before SMA was added to US recommendations in 2018 [29]. Implementation was also expedited by the fact that SCID screening had already been established, as SCID and SMA can be multiplexed together [20]. In New Zealand, it took five years from the addition of SCID to the screening panel to achieve nationwide implementation [10], whereas, in Catalonia, regional implementation of official NBS screening for SCID was achieved within 11 months from when the Department of Public Health communicated that SCID was approved for inclusion in its NBS programme [13]. However, the publication from Catalonia did not provide details on any preparatory work that may have taken place prior to this approval [13].

Although reporting challenges and facilitators affecting the implementation of SCID and SMA was not the primary focus of this review, several important themes emerged from the studies identified. Implementing NBS for SCID and SMA requires substantial laboratory infrastructure, including new equipment, sufficient and dedicated workspace, IT systems, and personnel with expertise in molecular testing [10,12,19,20,24]. In the US, the limited availability of experts in immunodeficiency for diagnosis and treatment was flagged as a key challenge [23]. Given this, the recruitment of clinical and laboratory personnel with the necessary skillset may delay implementation timelines. Developing screening, diagnostic and treatment algorithms that account for the severe and time-sensitive presentation of these conditions is essential [16,19,20]. Complex scenarios—such as cases where parents refuse treatment—should also be considered in advance [24]. Premature and low-birth-weight infants often yield out-of-range results and require modified algorithms and follow-up protocols [10,20]. Robust information and communication technology systems are vital for supporting sample tracking within NBS to ensure that newborns who require further testing are not missed [12]. Ensuring quality assurance and clear governance structures is essential to ensure the success of expanded NBS programmes [10,23,24]. International comparisons to evaluate long-term treatment outcomes and compare screening metrics are important given the rarity of these conditions; however, variability in classifications across regions and over time presents significant challenges [10,12,23].

### 4.2. Implications for Policy, Practice and Research

In the Irish context, implementing NBS for SCID and SMA is a policy and practice priority. This review highlights that establishing new bloodspot screening programmes in other jurisdictions typically took between two and six years.

From a research perspective, most of the literature screened focused on diagnostic and treatment pathways for the screened conditions or pilot studies, rather than on the implementation of national, regional, or state-level NBS programmes. There is a clear need for more applied implementation research, particularly studies that detail the practical steps required to achieve national, regional or state-level programme coverage, establish robust laboratory processes and elucidate how programmes progress from legislation to implementation. Such work could significantly accelerate and standardise the adoption of expanded NBS across countries. The use of standardised research reporting checklists, such as the Standards for Reporting Implementation Studies (StaRI) in implementation research, could enhance the utility of future implementation research [30].

### 4.3. Limitations

As mentioned previously, none of the studies in this review provided granular, step-by-step timelines for the implementation of expanded NBS programmes, limiting our ability to identify rate-limiting steps or dependencies. Regarding methodology, search, screening, article selection, data extraction and synthesis were conducted by single reviewers, which may introduce bias. Second, our search may not include all conditions for which it is possible to screen for in neonates. Third, we only searched two databases; however, we supplemented this with hand-searching six months of publications within the International Journal of Neonatal Screening and grey literature searches on US and UK websites. Lastly, our searches were restricted to English-language studies due to resource constraints within the research team.

## 5. Conclusions

Internationally, the implementation of new screening programmes for SCID and SMA typically takes between two and six years. There is a need for more applied implementation research detailing the processes involved in expanding NBS programmes.

## Figures and Tables

**Table 1:** Studies reporting timeframes taken to implement additional conditions (SCID, SMA or multiple) into national, regional or state-level NBS screening programmes published 2015–2025.

Author, Year	Country/Region	Study Design	Implementation Steps Reported	Time Taken to Implement (and How Defined)
*SCID*				
Heather et al., 2022 [10]	New Zealand	Report documenting the New Zealand experience and SCID screening performance	○ 2013: application to add SCID to the screening panel made by the national paediatric immunology service and reviewed by the National Screening Unit (NSU). ○ 2013: application approved ○ 2013: NSU commissioned a cost-utility study which showed cost per QALY was similar to other healthcare interventions. ○ 2015: A bid for government funding was successful. ○ December 2017: implemented within the national newborn screening programme (note did not conduct a pilot study but monitored first 3 years of programme (2017–2020) closely)	Approximately 5 years from addition to screening panel to national programme.
Strand et al., 2020 [11]	Norway	Prospective pilot research project performed in parallel with retrospective study	○ September 2015–December 2017: prospective pilot where newborns born in 6 selected hospitals were offered SCID screening. In parallel, a retrospective study using samples from patients with primary immunodeficiencies was performed to evaluate methods, establish cut-off levels, determine methodological sensitivity and specificity and explore the utility and efficacy of 2nd tier next generation sequencing (NGS) integrated in NBS. ○ January 2018: all newborns in Norway offered NBS for SCID.	Approximately 2.5 years from pilot to national programme.
Speckman et al., 2023 [12]	Germany	Report evaluating the German TREC-NBS process and discussion of remaining health, political and structural challenges 2.5 years after introduction	○ 2009–2019: preceding political process * ○ February 2019: announcement by the Gemeinsamer Bundesausschuss that TREC- based NBS was to be introduced. ○ Joint quality criteria were defined by the Working Party for Paediatric Immunology, the German Societies for Newborn Screening and for Child and Adolescent Medicine, and the German Society for Paediatric Haematology and Oncology. ○ August 2019: prospective nationwide TREC-based NBS was introduced. * no further details were provided on the political process in the paper	Approximately 11 years from initiation of political process to national programme.
Argudo-Ramírez et al., 2021 [13]	Catalonia	Report of the first three and a half years of experience with NBS for SCID	○ September 2016: the Department of Public Health of the Catalonian Government approved the inclusion of SCID in its NBS programme. ○ January 2017: six months prospective implementation pilot study undertaken to validate the approach. ○ July 2017: NBS for SCID officially started in Catalonia	Approximately 11 months from governmental approval to regional programme.
Göngrich et al., 2021 [14]	Sweden	Report of first year of SCID NBS	○ November 2013–November 2016: pilot study in the County of Stockholm. ○ August 2019: SCID introduced into national NBS programme.	Approximately 7 years from pilot to national programme.
Ricci et al., 2024 [15]	Tuscany	Retrospective single-center study	○ 2014–2018: pilot study in Florence ○ 2018: NBS Expanded Strategy implemented in Tuscany	Approximately 5 years from pilot to regional programme.
*SMA*				
Muller-Felber et al., 2023 [16]	Germany	Article describing implementation process, organisational requirements, challenges and timeframes.	○ January 2018–September 2021: pilot projects. ○ October 2021: included in the general NBS programme.	Approximately 3.8 years from pilot to national programme.
Boemer et al., 2021 [24]	Southern Belgium	Article describing lessons learned during implementation.	○ 2018–2021: pilot programmes ○ 1 March 2021: pilots transitioned into official programme run by public health service in Southern Belgium.	Approximately 4 years from pilot to regional programme.
Vrščaj et al., 2024 [18]	Worldwide	Survey of experts in 143 countries in 2023.	From pilot to national programme * ○ Belgium: March 2018–2022 ○ Czech Republic: January 2022–2024 ○ Estonia: July 2022–2024 ○ Germany: January 2018–July 2021 Poland: April 2021–March 2022 ○ Portugal: October 2022–2024 ○ Slovakia: October 2022–2024 ○ US: January 2016–July 2018 * Only countries with reported newborn screening coverage of 91–100% and known implementation timeframes were extracted.	From pilot to national programme: ○ Belgium: 4 years ○ Czech Republic: 2 years ○ >Estonia: 2 years ○ Germany: 3.5 years Poland: 11 months ○ Portugal: 2 years ○ Slovakia: 2 years ○ US: 2.5 years
*Multiple*				
Singh et al., 2023 [20]	US	A retrospective analysis of the implementation through December 2022 of the six conditions that were added to the Recommended Uniform Screening Panel (RUSP) during 2010–2018	○ May 2010–July 2018: six new conditions added to the RUSP: SCID (May 2010), critical congenital heart disease (CCHD, September 2011), glycogen storage disease type II (Pompe, March 2015), muco-polysaccharidosis type I (MPS I, February 2016), X-linked adrenoleukodystrophy (X-ALD, February 2016) and SMA (July 2018)	From addition to RUSP to state-level implementation (average taken per programme) ○ SCID: 4.3 years ○ CCHD: 2.7 years ○ Pompe: 4 years ○ MPS I: 3.2 years X-ALD: 3.5 years ○ SMA: 2.1 years
Belaramani et al., 2024 [21]	Hong Kong	Retrospective review	○ 2013: a group was set up to review the evidence for the expansion of NBS for inborn errors of metabolism (NBSIEM) ○ 2015: a task force was established to plan and prepare for the implementation of a pilot study for NBSIEM ○ 2015–2017: pilot programme of NBSIEM. ○ 2020: Phased implementation of NBSIEM in all birthing hospitals within the public healthcare system, with completion in October 2020. Of note, the public healthcare system covers approximately 36.3% of live births.	Approximately 6 years from pilot to national programme within public maternity hospitals
Lampret et al., 2020 [22]	Slovenia	Description of the extended NBS program.	○ 2014–2016: pilot study to estimate the incidences of inborn errors of metabolism) and support a large NBS programme expansion (previously had only screened for two condition). ○ September 2018–2019: the expanded NBS programme, including 17 additional metabolic conditions was gradually introduced to maternity wards over the first year.	Approximately 6 years from pilot to nationwide introduction.

**Table 2:** Grey literature on timelines associated with the implementation of newborn screening for SCID and SMA.

Author, Title, Year	Country	Key Findings
Secretary’s Advisory Committee on Heritable Disorders in Newborns and Children, Newborn Screening for Severe Combined Immunodeficiency Disorder (2011) [23].	US	SCID added to Recommended Uniform Screening Panel (RUSP) in 2010. In 2011, six had state-wide screening in place, one had partial screening, two had targeted pilots, ten had screening approved, twenty-eight were fact-finding and four were reliant on regional partners for NBS.
Kemper et al., Review of Newborn Screening Implementation for Spinal Muscular Atrophy Final Report (2020) [19].	US	SMA added to the RUSP in 2018. In 2018, two states offered universal NBS for SMA. By 24 May 2020 states had done so and 10 more planned to in the next year. At the time of addition to the RUSP, most states reported that it would take 1–3 years to gain approval to screen for SMA, then 1–3 years to secure funds.

**Table 3:** Challenges and facilitators encountered during implementation of newborn bloodspot screening for SCID and SMA.

Author, Year	Country/ Region	Challenges	Facilitators
*SCID*			
Heather et al., 2022 [10].	New Zealand	Dealing with the molecular technology and a test with non-linear results was challenging; appropriate lab space with agreed workflows is required. Screening pre-term, low-birthweight or otherwise unwell babies is different than term, healthy infants; there was a thirty-five fold increase in out of range results observed from samples collected in neonatal intensive care units relative to those in the community. Comparing screening metrics between programmes who use similar or common terms but defining them slightly differently; for example, using normal and abnormal values to distinguish between positive and negative screen results, versus defining a positive screen as any result that requires further action.	Close clinical involvement during the implementation process. Availability of a paediatric immunologist, to input on screening results, quality and algorithms. An external quality control scheme (provided by the Centre for Disease Control and Prevention) was important to validate quality of testing.
Singh et al., 2023 [20].	US	First condition that needed molecular technology for first-tier testing in NBS; required expanded lab capabilities, new equipment and agreed unidirectional workflows. Time and resources needed to determine biomarker thresholds for screening and to develop implementation plans. Modified screening algorithms may be needed for pre-term births. Staff hiring and training also needed to ensure competency in molecular technology.	Availability of qPCR and molecular analysis for first-tier screening lowered costs. FDA approved a commercially available kit for SCID screening in 2014.
Speckman et al., 2023 [12].	Germany	Technical issues in sample processing and pipetting: plates containing DBS-DNA eluate were overfilled, increasing the risk of spillage and potential cross-contamination. Absence of a NBS tracking system; this led to failures in recognising when a second sample was not obtained from a child whose initial TREC-NBS was inconclusive and later presented with fatal malignant lymphoma. Updates to diagnostic criteria.	Confirmatory testing was largely performed at specialised immunological institutions. A newly founded network of Combined Immunodeficiency Clinics and Centres and a scientific medical society supported timely initiation of protective measures and treatment of identified cases.
Secretary’s Advisory Committee on Heritable Disorders in Newborns and Children, 2011 [23].	US	Budgetary concerns (high cost estimates for technology infrastructure). Lack of widespread availability in experts in immunodeficiency for diagnosis and treatment.	Very accurate molecular methods were developed and validated. Model protocols for screening were used. An international database to assess lab performance and participation in a national quality assurance programme enabled real-time quality improvement. Sharing of expertise and lessons learned enabled timely management of positive screens and refinements of screening processes.
*SMA*			
Muller-Felber et al., 2023 [16].	Germany	Communication of screening results was delayed in a number of cases due to issues such as incorrect details (phone and address) on the screening card, and parents with hearing impairments who needed to be contacted in writing. Uncertainty about which centre would provide further care for children identified via NBS resulted in delayed presentation. Organisational structures needed to support cooperation between neuromuscular centres, maternity care and screening labs must be established. Managing false positive results appropriately; clear need for coordination between screening laboratories and treatment centres.	Early involvement of all stakeholders (patient advocacy groups and professional organisations) in the field was important. Easily available information for families online in many languages.
Boemer et al., 2021 [24].	Southern Belgium	Managing situations where parents refuse treatment.	Involvement of all from the beginning of the project and throughout was key to support transition from the trial project to governmental public health program. A clear governance structure supported the building of a strong partnership between pilot study leaders, regional agencies and NBS centres. Public involvement helped develop support politically. Acquisition of a dedicated qPCR instrument and hiring a lab technician to specifically focus on SMA screening supported scale-up.
Kemper et al., 2020 [19].	US	Deciding whether and how to include testing for SMN2 copy numbers. Follow up with carriers if detected. Availability of expert clinical care after a positive screen.	Ability to multiplex SMA screening with SCID (which was already recommended in every state) meant no additional equipment was needed. The experience states had with molecular testing for SCID was directly transferable to SMA, once procedural and validation details of multiplexing SMA into the SCID assay was optimised. Gold-standard genetic testing approaches have high throughput applications; quantitative polymerase chain reaction (qtPCR) is the preferred method in the US. Screening results only report that there is an absence of SMN1 exon 7 in both alleles; this simplifies interpretation and reporting of screening tests by not identifying carriers.
Vrščaj et al., 2024 [18].	Worldwide	Challenges in collaboration with authorities (N = 6). Lack of workforce resources (N = 3). Bureaucracy (N = 4). Concerns about genetic screening (N = 2). Academic conflicts (N = 2). Financial constraints (N = 2). Limited SMA awareness (N = 1). Inequality between regions (N = 1).	Patient associations (N = 7). Multi-level cooperation (N = 7). Sharing experiences with other jurisdictions (N = 6). Pilot studies (N = 4). NBS for metabolic diseases already established (N = 4). Pharmaceutical or other private company support (N = 4). Access to disease-modifying treatment (N = 3). Economic analyses (N = 2). Media coverage (N = 1). Governmental support (N = 1).
Singh et al., 2023 [20].	US	Initiation of treatment is time-sensitive. Whether and how to include additional testing for SMN2 copy number, which requires a separate assay.	Ability to screen for SMA and SCID at the same time in the same test and workflow.

## Data Availability

No new data were created or analysed in this study. Data sharing is not applicable to this article.

## References

[1] Health Service Executive (HSE) (2025). Heel Prick Screening. https://www2.hse.ie/conditions/heel-prick-screening/.

[2] Health Information and Quality Authority (2023). Health Technology Assessment of the Addition of Severe Combined Immunodeficiency (SCID) to the National Newborn Bloodspot Screening Programme. https://www.hiqa.ie/sites/default/files/2023-01/HTA_addition_of_SCID_to_NNBSP_Jan-2023.pdf.

[3] Health Information and Quality Authority (2023). Health Technology Assessment of the Addition of Spinal Muscular Atrophy (SMA) to the National Newborn Bloodspot Screening Programme. https://www.hiqa.ie/sites/default/files/2023-11/Addition-of-SMA-to-NNBSP.pdf.

[4] National Screening Advisory Committee (NSAC) (2024). Expansion of the National Newborn Bloodspot Screening (NBS) Programme. https://assets.gov.ie/static/documents/expansion-of-the-national-newborn-bloodspot-screening-nbs-programme.pdf.

[5] Tricco A.C., Lillie E., Zarin W., O’Brien K.K., Colquhoun H., Levac D., Moher D., Peters M.D.J., Horsley T., Weeks L. (2018). PRISMA extension for scoping reviews (PRISMA-ScR): Checklist and explanation. Ann. Intern. Med..

[6] Weill Cornell Medicine Samuel J. Wood Library (2025). Systematic Reviews: Scoping Reviews. https://med.cornell.libguides.com/systematicreviews/scopingreviews.

[7] Illinois Department of Public Health Genetics and Newborn Screening Tandem Mass Spectrometry Newborn Screening Information for Physicians and Other Health Care Providers. https://www.idph.state.il.us/HealthWellness/msmsfaq.htm#:~:text=This%20technology%2C%20tandem%20mass%20spectrometry,and%20fatty%20acid%20oxidation%20disorders.

[8] Irwin A.N., Rackham D. (2017). Comparison of the time-to-indexing in PubMed between biomedical journals according to impact factor, discipline, and focus. Res. Soc. Adm. Pharm..

[9] (2022). Covidence Systematic Review Software, Veritas Health Innovation, Melbourne, Australia. https://www.covidence.org/.

[10] Heather N., de Hora M., Brothers S., Grainger P., Knoll D., Webster D. (2022). Introducing Newborn Screening for Severe Combined Immunodeficiency—The New Zealand Experience. Int. J. Neonatal Screen..

[11] Strand J., Gul K.A., Erichsen H.C., Lundman E., Berge M.C., Trømborg A.K., Sørgjerd L.K., Ytre-Arne M., Hogner S., Halsne R. (2020). Second-Tier Next Generation Sequencing Integrated in Nationwide Newborn Screening Provides Rapid Molecular Diagnostics of Severe Combined Immunodeficiency. Front. Immunol..

[12] Speckmann C., Nennstiel U., Hönig M., Albert M.H., Ghosh S., Schuetz C., Brockow I., Hörster F., Niehues T., Ehl S. (2023). Prospective Newborn Screening for SCID in Germany: A First Analysis by the Pediatric Immunology Working Group (API). J. Clin. Immunol..

[13] Argudo-Ramírez A., Martín-Nalda A., González de Aledo-Castillo J.M., López-Galera R., Marín-Soria J.L., Pajares-García S., Martínez-Gallo M., García-Prat M., Colobran R., Riviere J.G. (2021). Newborn screening for SCID: Experience in Spain (Catalonia). Int. J. Neonatal Screen..

[14] Göngrich C., Ekwall O., Sundin M., Brodszki N., Fasth A., Marits P., Dysting S., Jonsson S., Barbaro M., Wedell A. (2021). First year of TREC-based national SCID screening in Sweden. Int. J. Neonatal Screen..

[15] Ricci S., Guarnieri V., Capitanini F., Pelosi C., Astorino V., Boscia S., Calistri E., Canessa C., Cortimiglia M., Lippi F. (2024). Expanded Newborn Screening for Inborn Errors of Immunity: The Experience of Tuscany. J. Allergy Clin. Immunol. Pract..

[16] Müller-Felber W., Blaschek A., Schwartz O., Gläser D., Nennstiel U., Brockow I., Wirth B., Burggraf S., Röschinger W., Becker M. (2023). Newbornscreening SMA—From Pilot Project to Nationwide Screening in Germany. J. Neuromuscul. Dis..

[17] Boemer F., Caberg J.H., Dideberg V., Dardenne D., Bours V., Hiligsmann M., Dangouloff T., Servais L. (2019). Newborn screening for SMA in Southern Belgium. Neuromuscul. Disord..

[18] Vrščaj E., Dangouloff T., Osredkar D., Servais L. (2024). Newborn screening programs for spinal muscular atrophy worldwide in 2023. J. Neuromuscul. Dis..

[19] Kemper A.R., Ream M.A., Lam K.K. (2020). Review of Newborn Screening Implementation for Spinal Muscular Atrophy Final Report. Prepared for The Health Resources and Services Administration (HRSA) Maternal and Child Health Bureau. https://www.hrsa.gov/sites/default/files/hrsa/advisory-committees/heritable-disorders/reports-recommendations/sma-nbs-implementation-report.pdf.

[20] Singh S., Ojodu J., Kemper A.R., Lam W.K.K., Grosse S.D. (2023). Implementation of Newborn Screening for Conditions in the United States First Recommended during 2010–2018. Int. J. Neonatal Screen..

[21] Belaramani K.M., Chan T.C.H., Hau E.W.L., Yeung M.C.W., Kwok A.M.K., Lo I.F.M., Law T.H.F., Wu H., Na Wong S.S., Lam S.W. (2024). Expanded Newborn Screening for Inborn Errors of Metabolism in Hong Kong: Results and Outcome of a 7 Year Journey. Int. J. Neonatal Screen..

[22] Repič Lampret B., Remec Ž.I., Drole Torkar A., Žerjav Tanšek M., Šmon A., Koračin V., Čuk V., Perko D., Ulaga B., Jelovšek A.M. (2020). Expanded newborn screening program in Slovenia using tandem mass spectrometry and confirmatory next generation sequencing genetic testing. Zdr. Varst..

[23] Secretary’s Advisory Committee on Heritable Disorders in Newborns and Children (2011). Newborn Screening for Severe Combined Immunodeficiency Disorder. https://www.hrsa.gov/sites/default/files/hrsa/advisory-committees/heritable-disorders/reports-recommendations/newborn-screening-scid-report.pdf.

[24] Boemer F., Caberg J.H., Beckers P., Dideberg V., di Fiore S., Bours V., Marie S., Dewulf J., Marcelis L., Deconinck N. (2021). Three years pilot of spinal muscular atrophy newborn screening turned into official program in Southern Belgium. Sci. Rep..

[25] UK National Screening Committee (2023). Antenatal and Newborn Screening Programme: Spinal Muscular Atropy. https://view-health-screening-recommendations.service.gov.uk/sma/.

[26] UK National Screening Committee (2017). Newborn Screening Programme: SCID. https://view-health-screening-recommendations.service.gov.uk/scid/.

[27] UK National Screening Committee (2024). SCID Screening In-Service Evaluation: Analysis of Results Under Way. https://nationalscreening.blog.gov.uk/2024/05/21/scid-screening-in-service-evaluation-analysis-of-results-under-way/.

[28] UK National Screening Committee (2024). SMA Screening In-Service Evaluation Update. https://nationalscreening.blog.gov.uk/2024/07/10/sma-screening-in-service-evaluation-update/.

[29] Lee F.K., Greene C., Mercer K., Taylor J., Yazdanpanah G., Vogt R., Lee R., Cuthbert C., Cordovado S. (2024). CDC’s Laboratory Activities to Support Newborn Screening for Spinal Muscular Atrophy. Int. J. Neonatal Screen..

[30] Pinnock H., Barwick M., Carpenter C.R., Eldridge S., Grandes G., Griffiths C.J., Rycroft-Malone J., Meissner P., Murray E., Patel A. (2017). Standards for Reporting Implementation Studies (StaRI): Explanation and elaboration document. BMJ.

